# A hybrid residue based sequential encoding mechanism with XGBoost improved ensemble model for identifying 5-hydroxymethylcytosine modifications

**DOI:** 10.1038/s41598-024-71568-z

**Published:** 2024-09-06

**Authors:** Islam Uddin, Hamid Hussain Awan, Majdi Khalid, Salman Khan, Shahid Akbar, Mahidur R. Sarker, Maher G. M. Abdolrasol, Thamer A. H. Alghamdi

**Affiliations:** 1 Department of Computer Science, Abdul Wali Khan University, Mardan, Pakistan; 2 Department of Computer Science, Muslim Youth University, Islamabad, Pakistan; 3 Department of Computer Science and Artificial Intelligence, College of Computing, Umm Al-Qura University, Makkah, 21955 Saudi Arabia; 4 Institute of Fundamental and Frontier Sciences, University of Electronic Science and Technology of China, Chengdu, 610054 China; 5 Institute of Visual Informatics, Universiti Kebangsaan Malaysia, Bangi, 43600 Selangor Malaysia; 6 Universidad de Diseño, Innovación y Tecnología, UDIT, Av. Alfonso XIII, 97, 28016 Madrid, Spain; 7 Institute of Sustainable Energy, Universiti Tenaga Nasional, Kajang, 43000 Malaysia; 8 Wolfson Centre for Magnetics, School of Engineering, Cardiff University, Cardiff, CF24 3AA UK; 9 Electrical Engineering Department, Faculty of Engineering, Al-Baha University, Al-Baha, 65779 Saudi Arabia

**Keywords:** RNA modifications, 5-Hydroxymethylcytosine, TET enzyme, Machine learning, Cancer, Cardiovascular diseases, XGBoost, Cell biology, Computational biology and bioinformatics, Systems biology

## Abstract

RNA modifications play an important role in actively controlling recently created formation in cellular regulation mechanisms, which link them to gene expression and protein. The RNA modifications have numerous alterations, presenting broad glimpses of RNA’s operations and character. The modification process by the TET enzyme oxidation is the crucial change associated with cytosine hydroxymethylation. The effect of CR is an alteration in specific biochemical ways of the organism, such as gene expression and epigenetic alterations. Traditional laboratory systems that identify 5-hydroxymethylcytosine (5hmC) samples are expensive and time-consuming compared to other methods. To address this challenge, the paper proposed XGB5hmC, a machine learning algorithm based on a robust gradient boosting algorithm (XGBoost), with different residue based formulation methods to identify 5hmC samples. Their results were amalgamated, and six different frequency residue based encoding features were fused to form a hybrid vector in order to enhance model discrimination capabilities. In addition, the proposed model incorporates SHAP (Shapley Additive Explanations) based feature selection to demonstrate model interpretability by highlighting the high contributory features. Among the applied machine learning algorithms, the XGBoost ensemble model using the tenfold cross-validation test achieved improved results than existing state-of-the-art models. Our model reported an accuracy of 89.97%, sensitivity of 87.78%, specificity of 94.45%, F1-score of 0.8934%, and MCC of 0.8764%. This study highlights the potential to provide valuable insights for enhancing medical assessment and treatment protocols, representing a significant advancement in RNA modification analysis.

## Introduction

RNA, the primary molecule that is the center of cellular processes, is indispensable for synthesizing proteins and transmitting genetic information. Its presence in all living beings stresses that is where the real strength lies^1^. Composed of a complicated molecular assembly, RNA carries hereditary information and performs the function of a biological catalyst. Moving further from its role inside the cells, RNA functions and transfers genome information via certain viruses^2^. Among RNA modifications, the essential role of this process is strengthened, and currently, more than 100 RNA modifications opening up new regulation possibilities are identified^3^. However, mRNA demonstrates N6-Methyladenosine and N7-Methylguanosine modifications, regulatory tools in mRNA’s different phases. Transferred RNA (tRNA) and ribosomal RNA (rRNA) present post-translational modifications like 5mC and N1MeA, which contribute to their function^4,5^. Besides forming 5hmC, a TET oxidation product, this further complicates the issue of RNA modifications^6^. The Appreciation of diverse RNA actions is vital to establish these roles, as they have far-reaching implications. All mRNA, tRNA, and rRNA molecules are involved in a complex interplay of molecular modifications that organize cellular processes, giving rise to cell-level changes in gene expression and protein synthesis and, thus, influencing the organism’s functioning at the whole level^7,8^. A presentation of the fine-tunings of RNA modifications will further propel our knowledge of biological systems, ultimately leading to the creation of novel therapeutic approaches and revolutionary discoveries in molecular biology.

The 5hmC alteration was discovered, and the study that found the answer stemmed from the wheat seeds, exposing that life’s boundaries are not as restricted as people think. The empirical basis of this pivotal insight is stretched over species and fields of inquiry, thus a remarkable scope and magnitude^9^. The full scale of the influence of 5hmC variation shows the way and leads to the correct interpretation of genetic phenomena in the complicated fabric of genetics^10^. 5hmC modification deviates from the ratio in wheat seed and tissues of humans and mice performing RNA splicing, translation, and decay functions. It affects the process of gene expression. Thus, its role must be considered in understanding the mechanisms of epigenetic regulation and the use of eukaryotic genetic expression^11^. Furthermore, the 5hmC functional area native to man is crucial to discuss with a close connection to diseases like cancer, diabetes, and cardiovascular disease. Hence, it is essential to analyze 5hmC modifications in studying the complex human health system and establish the reason for this approach as a unique scientific contribution to medical sciences to enhance patient-tailored treatment^12^. The biochemical and chemical methods, i.e., LC–MS/MS, HPLC, and TLC, have been intensively developed to identify this new molecule (5hmC). This technique endorsed the accuracy of 5hmC alteration to an excellent level. Complementary PCR- and chromatography-based techniques are essential for resolving the problems emerging during 5hmC studies^13^. Combining different techniques, researchers are on their way to exploring 5hmC dynamics in biological processes, depicting, interpreting, and manipulating them. However, these methods have been established as accurate as the relatively complicated and protracted procedures for detecting 5hmC are performed accurately. The discovery of the 5hmC modification in wheat seeds has implications beyond transformation, revolutionizing multiple species across various biological domains. Moreover, further research is required due to its diverse impacts on genetic processes and its association with human diseases. This would help in the creation of new strategies for prevention and intervention. In addition to that, the complex techniques applied in locating 5hmC help to solve the hidden relationship, though the limitations in terms of time and cost are need to consider.

Among the recent machine learning approaches to recognizing ephemeral 5-hydroxymethylcytosine (5hmC) sites, we highlight a growing number of studies. The first research to explore the machine-learning stage was presented by Liu et al.^14^ with the transition from traditional SVM algorithms to sequence-based methodology. The executed research included cutting-edge techniques that allowed for more efficient completion of the intended goals. This method also moved the scene of 5hmC classification many degrees away and presented us with the enormous unknown of the epigenetic world with its five-dimensional non-linear matrices. Ahmed et al.^15^ developed the iRNA5hmC-PS model employing the PSG k-mer technique, regarded as the most efficient feature extraction, and the Logistic Regression model, the most reliable classifier algorithm. Although their contributions are evident, let us not forget that learning app use will remain reliant on traditional approaches. The striking commonalities in such models make it difficult to find proper and exact ways of forecasting 5hmC sequences. Therefore, its identification requires a lot of human effort and the application of computer resources to extract the necessary features. Recently, a breakthrough was made by Ali et al.^16^, they successively constructed a new model called iRhmC5XGB5 that switches from the commonly-used deep neural network solutions to adopt the ultra-efficient and modern XGBoost algorithm for recognizing 5hmC. This autonomous design is proof of concept and highlights XGBoost role amongst the leading algorithms. One-hot-encoding-based features were provided to train the XGBoost model, and the outcome sample stands out for high performance. Delving into the innovation, the iRhmC5XGB5 model presents a different approach and new abilities that are separated from the conventional neural network methodologies. Hence, XGBoost was considered an effective tool in generalize model training, but it also competes very successfully among other methods in revealing the intricate connection between epigenetic marks and other key biological variables.

Based on recent empirical studies, this study will introduce an intelligent and robust machine learning model, adapting and adjusting Chou’s 5 steps, by extracting the discriminative features with the XGBoost model for predicting 5-hydroxymethylcytosine (5hmC) modification. Our modified machine learning model, not only directly (XGB5hmC) or indirectly, facilitates the initiator of the original machine learning. XGB5hmC uses seven feature extraction techniques that convert the RNA sequences into feature vectors. All the extracted feature vectors are finally combined into a fused feature vector. Additionally, the SHAP analysis-based high contributory features were selected by choosing prominent features and removing inessential and redundant information. Finally, among the several learning models, the XGBoost training model using a tenfold cross-validation test is selected as a training model due to its high training capabilities.

The main innovation of the paper is the following:

- Firstly, this paper employed Chou’s 5-step procedure by incorporating different structured and sequential-based feature encoding schemes to predict 5-hydroxymethylcytosine (5hmC) modification prediction.
- Secondly, the novel SHAP interpolation-based feature selection approach was employed to choose highly relevant and discriminative features from the extracted set.
- Thirdly, an improved XGBoost learning model was proposed to improve the predicted outcomes and robustness of 5hmC modification, surpassing traditional machine learning techniques.
- Finally, the proposed optimal set-based training model is tested via an independent dataset to thoroughly evaluate model overfitting and its generalization.

The remainder of this paper is organized in the following order: Section “Literature review” of the paper describes the related work. Section “Material and methods” describes the suggested model materials and methods in detail. Section “Performance evaluation matrix” addresses the paper’s experimental results, and Section “Experimental results and analysis” provides the paper conclusion and future work.

## Literature review

In recent years, there has been a rapid advancement in the field of bioinformatics related to epigenetics. This progress includes the application of machine learning techniques to identify and explain the structures of 5-hydroxymethylcytosine (5hmC) sites, which are well-known yet significant epigenetic modifications that play a crucial role in the regulation of gene expression. The study of gene expression and its regulation by epigenetic mechanisms further demonstrates the intricate relationship between these processes in biological systems. This understanding aids in identifying diseases that warrant further investigation and determining the most effective treatment options.

A novel method introduced by^14^, an SVM algorithm, is integrated with an innovative one-level feature extraction method. Their proposed model achieved high accuracy in 5hmC localization through the use of cutting-edge machine learning techniques, providing new insights into epigenetics. Similarly^15^, introduced iRNA5hmC-PS, a hybrid model combining k-mer-based features of the PSG and LR features. Despite recent achievements, traditional learning models still face challenges in sequencing and quantification of 5hmC due to difficulties in feature extraction and unreliable experimental design methods. To address these issues^16^, proposed a new strategy called iRhmC5CNN, which classifies the 5hmC sequence detection problem into several tasks and employs convolutional neural networks (CNNs) for each task. The CNN model, designed and tested on a deep learning dataset using deep and one-hot encoding methods, demonstrated that deep learning can significantly benefit epigenetic research.

Further improvements have been identified, suggesting that more promising results are achievable. However, despite significant progress, many challenges remain, and additional efforts are needed to develop deep learning algorithms for detecting and predicting 5hmC sites. These algorithms can elucidate the complex mechanisms of genomic regions involved in 5hmC modification. The simpler design and faster generalization ability are crucial considerations when implementing models in epigenetic studies. Another recent model, Deep5hmC, developed by^17^, focuses on using deep neural networks and hybrid features for the accurate detection of 5hmC modifications. Recognizing the importance of RNA modification in gene regulation and epigenetic modification, they aimed to overcome the labor-intensive and costly challenges of previous RNA detection techniques. Their approach combines seven unique feature extraction methods with several machine learning procedures, including Random Forest, Naive Bayes, Decision Tree, and Support Vector Machine. The model achieved high accuracy, surpassing prior models by 7.59% through K-fold cross-validation. This advancement shows how Deep5hmC can improve the early detection of cancer and cardiovascular diseases, marking a significant step forward in RNA modification analysis. Combining multi-data and enhancing the ensemble learning setup can play a vital role in the accuracy and reliability of predictive models for 5hmC sites. Computational biologists, bioinformaticians, and experimental biologists need to advance machine-learning algorithms based on biological experiments and verify their predictions through experimentation. The new machine learning technology transforming the epigenetic field has fostered a genuine interest in the complexity of epigenetic control and its impact on cell functions and human health. Researchers can leverage both computational approaches and experimental tools to effectively understand how 5hmC modification influences gene expression regulation, development, and disease progression.

## Material and methods

**Fig. 1:**
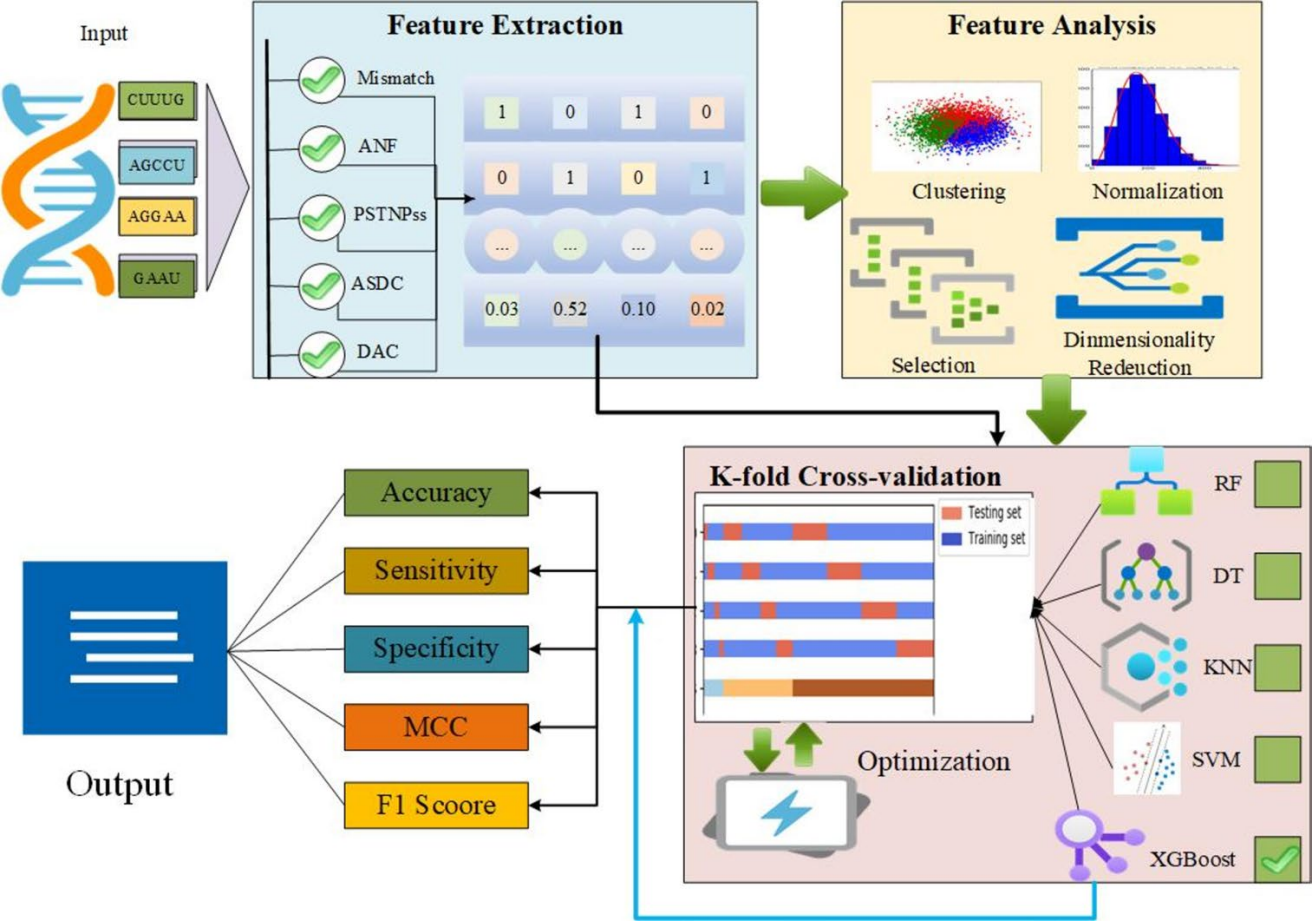
Predictive Architecture for Identifying 5hmC Modifications in RNA Sequences. This figure depicts the architecture of the model used to predict 5-hydroxymethylcytosine (5hmC) modifications in RNA sequences. It outlines the steps from data input and feature extraction to the final machine learning model (XGBoost) used for prediction. The workflow highlights the integration of various computational techniques to identify 5hmC sites effectively**.**

The 5-methylcytidine-positioned RNA modification predictor involves several interrelated steps. It begins with extracting sequences from steady-state DNA (ssDNA) and applying feature extraction techniques. The extracted features are then analyzed and optimized using feature selection techniques. Lastly, Predictive modeling employs different performance assessment parameters such as accuracy, sensitivity, specificity, Matthew Correlation Coefficient, and precision-recall scores. The complete framework of the proposed methods model representing each phase of the model is provided in Fig. 1.

### Benchmark dataset

In bioinformatics and machine learning, the acquisition or selection of a valid benchmark dataset is an essential step for developing an intelligent computational model. The selection of a suitable benchmark has a high impact on the performance of a computational model. According to Chou’s comprehensive review^18,19^, a valid and reliable benchmark dataset is always required for the design of a powerful and robust computational model. Hence, in this paper, we used the same benchmark datasets that were used in^16^. The selected datasets can be expressed in mathematical form using Eq. (1).

1 $$R_{1} = R_{1}^{ + } + R_{1}^{ - }$$

where, ${R}_{1}$ represent the total number of 5hmC samples, ${R}_{1}^{+}$ represent the positive 5hmC samples and ${R}_{1}^{-}$ all negative 5hmC samples. Firstly, CD-HIT software was applied using a threshold value of 80% to eliminate high resemblance sequences. Secondly, we employed a random sampling technique to select the same number of positive samples as that of the negative samples to balance the benchmark dataset. Finally, we obtained a benchmark dataset that contained a total of 1324 sequences, of which 662 are 5hmC sequences and 662 are non-5hmC samples. To assess the generalization power of our proposed model, we employed an independent dataset. To create an independent test set, the original dataset is divided into two parts: 80% of the samples are allocated for training, while 20% are reserved for testing. The 20% used for testing is randomly selected to form an independent dataset. The training phase then utilizes the remaining 80% of the data. The independent dataset consists of 264 sequences, of which 132 are positive samples and 132 are negative samples.

### Feature extraction techniques

To generate prominent, reliable, and variant statistical-based discriminative descriptors, several feature encoding approaches have been utilized for the formulation of proteins, RNA, and DNA sequences^20^. The detailed overview of the proposed feature encoding schemes is presented in the below sections.

#### Mismatches

Mismatch^21^ calculates the occurrences of k-length neighboring nucleic acids that differ by at most m mismatches (m < k). The second step of the profile is the allowance for the maximum number of m mismatches instead of the sole occurrence analysis of k-mers. For a 3-length subsequence “AAC” and max one mismatch, we need to consider 3 cases, “–AC”, ”A–C” and “AA–”, “–” can be replaced by any nucleic acid residue. This descriptor is governed by two parameters.

K Represents the nearby nucleic acids or k-mers, which are considered "neighbors" in the analysis and *m* is the number of mismatches allowed. This threshold defines how flexible the matching process is^22^. A mismatching threshold is written as m, shortening the word inexact matching to match and formalized as in Eq. (2), the matching descriptor, labeled as $c_{i,j}$ describes cases when the ith class occurrences with j mismatches occur.

2 $$C_{k,m} = \left( {\sum\limits_{j = 0}^{m} {C_{1,j} ,} \sum\limits_{j = 0}^{m} {C_{2,j} , \ldots ,} \sum\limits_{j = 0}^{m} {C_{{4^{K} ,j}} } } \right)$$

where, C_i j_ represents the occurrences of i-th k-mer type, with j mismatches, $i = 1,2,3, \ldots ,4^{k}$;$j = 0,1,2,...,m$

#### Accumulated Nucleotide Frequency (ANF)

The Accumulated Nucleotide Frequency (ANF) method of encoding^23^ makes it possible to encode the sequence of each nucleus, as well as to take into account the nucleotide distribution within the sequence of the RNA^24^. The density $d_{i}$ of any nucleotide $s_{i}$ at position $i$ in the RNA sequence is computed using Eq. 4.

3 $$d_{i} = \frac{1}{{s_{i} }}\sum\limits_{j = 1}^{{s_{i} }} {f(s_{i} ),f(q) = \left\{ {_{0\,\,\,\,other\,\,case}^{{1\,\,\,\,\,ifs_{i} = q}} } \right\}}$$

Here, variable l denotes the resulting sequence length; $s_{i}$ it simply represents the last size $i_{prefixes} \left\{ {s_{1} ,s_{2} , \ldots ,s_{i} } \right\}$ in our sequence, and q is an element belonging to the set (A, C, G, U). Let us get in-depth with the sequence "UCGUUCAUGG". Starting with position 1, we have a density value of ‘U,’ which is 1, 0.5 for position 4, and 0.6 for position 5. Finally, 0.5 for position 8. ‘C’ carbon atoms have two times higher density when in position 2 instead of position 6, as there are 0.5 and 0.33, respectively, the ‘G’ nucleotide positions 3 and 9 counts for 0.33 and 0.22, respectively. Position 10 of the nucleotide seems to have the highest count, -0.3. The case of H2O at position 7 is ‘A,’ which has 0.14 density.

#### Position-specific trinucleotide propensity based on single-strand (PSTNPSS)

PSTNPss, which stands for Position-specific trinucleotide propensity based on single-strand, is a computational method developed to analyze the characteristics of single-stranded DNA or RNA. This approach, outlined in studies^25,26^, focuses on understanding the statistical properties of trinucleotides within biological sequences^27,28^. With 64 possible trinucleotides (AAA, AAC, AAG,…,U), PSTNPs aim to capture each trinucleotide’s position-specific propensity within a given sequence. To achieve this, PSTNPss utilizes a matrix representation, typically of dimensions 64 × (L-2), where L represents the sequence length in base pairs. Each cell in this matrix corresponds to a specific trinucleotide at a particular position within the sequence. By analyzing the frequency and distribution of trinucleotides across different positions, PSTNPs provide insights into the positional preferences of trinucleotides along the single-stranded DNA or RNA sequences. This approach enables researchers to uncover patterns and trends indicating functional or structural elements within the genetic material.

4 $$z = \left[ {\begin{array}{*{20}c} {z_{1,1} } & {z_{1.2} } & {...} & {z_{1,L - 2} } \\ {z_{2,1} } & {z_{2,2} } & {...} & {z_{2,L - 2} } \\ \vdots & \vdots & {...} & \vdots \\ {z_{64,1} } & {z_{64,2} } & {...} & {z_{64,L - 2} } \\ \end{array} } \right]$$

In the given formula:

5 $$z_{i,j} = F^{ + } (3mer_{i} |j) - F^{ - } (3mer_{i} |j)$$

- $i$ ranges from 1 to 64, representing the 64 possible trinucleotides.
- $j$, ranges from 1 to $L - 2$, where $L$ is the sequence length, indicating the positions within the sequence.
- $F^{ + } (3mer_{i} |j)\;and\;F^{ - } (3mer_{i} |j)$, denote the frequency of the ith trinucleotide (3meri) at the jth position in the positive $\left( {S^{ + } } \right)$ and negative $\left( {S^{ - } } \right)$ datasets.
- For instance, 3mer1 corresponds to AAA, 3mer2 corresponds to AAC, and so on, up to 3mer 64 features, which corresponds to TTT.

Therefore, the sample can be represented as follows:

6 $$s = \left[ {\theta_{1} ,\theta_{2} , \ldots ,\theta_{L - 2} } \right]^{T}$$

where $T$ denotes the transpose operator and $\phi_{u}$ is defined as follows:

7 $$\phi_{u} \left\{ {\begin{array}{*{20}c} {z_{1,u} } & {when\,N_{u} N_{u + 1} N_{u + 2} = AAA} \\ {z_{2,u} } & {when\,N_{u} N_{u + 1} N_{u + 2} = AAG} \\ {} & \vdots \\ {z_{64,u} } & {when\,N_{u} N_{u + 1} N_{u + 2} = UUU} \\ \end{array} } \right.$$

The PSTNP descriptor, utilizing a statistical approach based on single-stranded DNA or RNA characteristics, has been successfully applied in predicting DNA N4-methylcytosine sites^29^. It involves calculating the frequency of each trinucleotide at different positions in positive and negative datasets, representing them in a matrix format and has shown efficacy in its predictive capabilities.

#### Adaptive skip dipeptide composition (ASDC)

The Adaptive Skip Dipeptide Composition (ASDC) is an advanced form of dinucleotide composition designed to incorporate correlation information between adjacent and intervening residues^30^. In ASDC, the feature vector for a given sequence is represented as follows:

8 $$ASDC = (f_{v1} ,f_{v2} , \ldots ,f_{vns} )$$

The formula determines the calculation of ASDC:

9 $$f_{vi} = \frac{{\sum\limits_{g = 1}^{L - 1} {O_{i}^{g} } }}{{\sum\limits_{i = 1}^{16} {\sum\limits_{g = 1}^{L - 1} {O_{i}^{g} } } }}$$

where $f_{vi}$ represents the occurrence frequency of all possible dinucleotides with up to $\le L - 1$ intervening nucleotides. The ASDC descriptor has demonstrated successful applications in predicting anti-cancer peptides and cell-penetrating peptides^31^.

#### Dinucleotide-based auto covariance (DAC)

The Dinucleotide-based Auto Covariance (DAC) encoding^32,33^ measures the correlation of the same physicochemical index between two dinucleotides separated by a lag distance along the sequence^34,35^. The DAC can be calculated as:

10 $$DAC(u,lag) = \sum\limits_{i = 1}^{L - lag} {\left( {\frac{{\left[ {P_{u} (R_{i} R_{i + lag} ) - p^{\prime}_{u} } \right]\left[ {p_{u} (R_{i + lag} R_{i + 2\& *lag} ) - p^{\prime}_{u} } \right]}}{L - lag - 1}} \right)}$$

where $u$ is a physicochemical index, $L$ is the length of the nucleotide sequence, $P_{u} (R_{i} \;R_{i + 1} )$ is the numerical value of the physicochemical index $u$ for the dinucleotide $RiR_{i + 1}$ at position $i, P^{\prime}_{u}$, and is the average value for physicochemical index $u$ along the whole sequence:

11 $$P^{\prime}_{u} = \frac{{\sum {p_{u} (Ri R_{i + 1} )} }}{L - 1}$$

The dimension of the DAC feature vector is $N \times LAG$ N, and the number of physicochemical indices and LAG are the maximum lags $\left( {lag \, = \, 1, \, 2, \, ..., \, LAG} \right)$.

### Fused feature vector

In this model, we applied five different feature encodings such as Mismatches (MisM), ASDC, DAC, PSTNPSS, and ANF to capture the nucleotide-based features keeping their residue ordering information. Moreover, to generate the high discriminative model representing the multi-perspective features, we serially concatenated the extracted features to form an individual vector covering the weakness of the individual feature vector as follows:

12 $$Hybrid\_Vector = MisM \cup ASDC \cup DAC \cup ANF \cup PSTNPSS$$

### SHAP feature selection

It is not always straightforward to determine the biological significance of the selected descriptors. The machine learning algorithms are sometimes called “black boxes.” due to their complex inner structure. Discussions on data shape in machine learning refer to the structure or size of data groups used for various tasks. This aspect is crucial for data owners as they need to determine the dataset size and how to process it efficiently. Machine learning algorithms can exhibit differences in performance and usability depending on the dataset they are trained on. Understanding the data shape is essential for preprocessing steps such as splitting the data into training and testing sets, normalization, and feature selection. Properly shaping the data provides critical insights that guide decision-making, which is a key element in the data science pipeline. SHAPley Additive Explanations (SHAP) uses cooperative game theory to distribute credit among the contributions of input features in machine learning algorithms^36–38^. It assigns a specific quantitative value to each feature input and tells which predicts the value better. Measurement-wise, SHAP adds various contributions from the features of interest in and out of the model. Such difference shows the extent of an element’s influence on the result, and Eq. 14 shows the formal mathematical formulation. This Equation captures the incremental effect of adding feature $\text{i}$, to different subsets of features.

13 $$SHAP_{i} (x) = \phi_{i} = \sum\nolimits_{{s \subseteq N\backslash \{ i\} }} {\frac{\left| S \right|(\left| N \right| - \left| S \right| - 1)}{{\left| N \right|}}} \left[ {f(S \cup \left\{ i \right\}) - f(S)} \right]$$

where:

- $\phi_{i}$, represents the SHAP value for the feature $i$.
- $N$, is the set of all features.
- $S$, is a subset of features excluding i.
- $f\left( S \right)$ is the model’s prediction given features in S.
- $f(S \cup \left\{ i \right\})$ is the model’s prediction given features in S plus feature $\text{i}.$

**Fig. 2:**
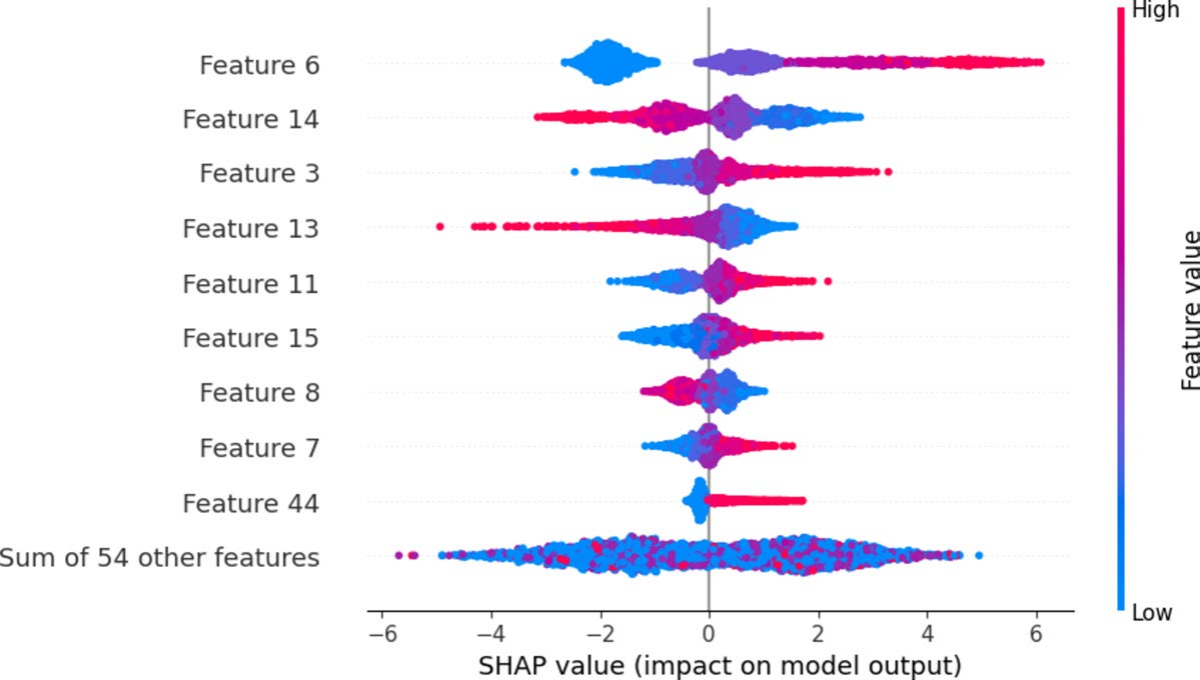
SHAP-Based Feature Selection on the Hybrid Features. This figure illustrates the SHAP analysis used to select important features from the hybrid feature set, identifying those that contribute most significantly to the model’s predictions.

Figure 2, show the main features selected for this study. Every row in these charts is a different feature, and each dot shows the SHAP value for that feature in a specific example. Red dots mean the feature’s value is high, and blue dots mean it is low. The horizontal axis shows the SHAP values, which indicate how much each feature influences the model’s predictions. A positive SHAP value means the feature increases the chances of AVPs (presumably a positive outcome).

**Fig. 3:**
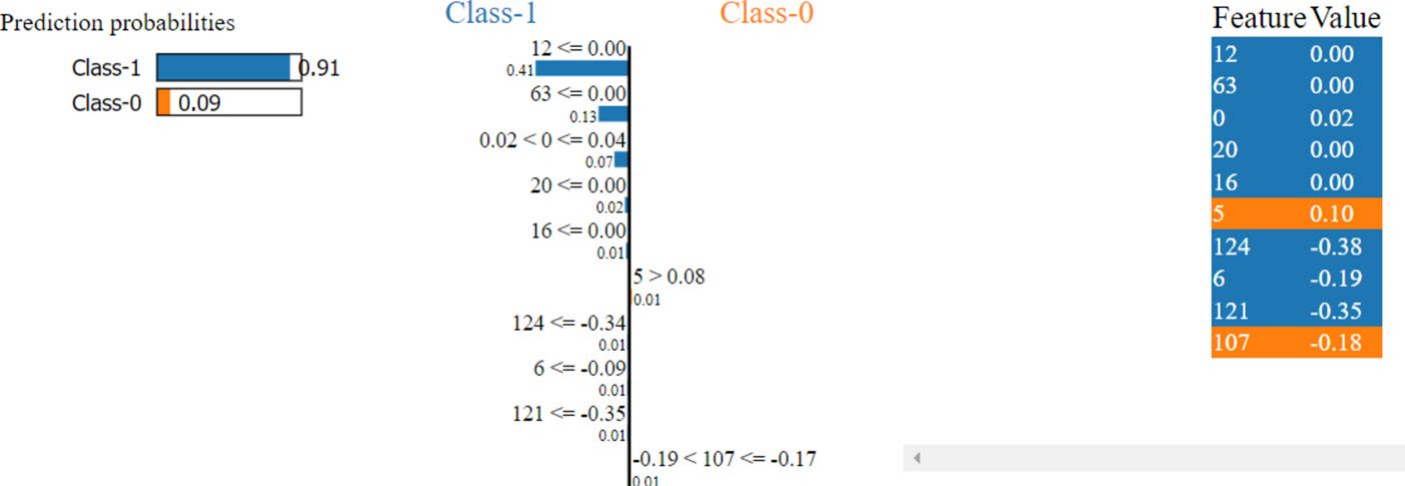
LIME analysis on randomly selected instances after SHAP feature selection*.* This figure presents the LIME analysis applied to randomly selected instances, following SHAP-based feature selection, to provide interpretability of the model’s predictions by highlighting the contribution of individual features.

In contrast, a negative value means it increases the chances of non-AVPs (a negative outcome). In our study, we tested different groups of features of varying sizes. However, we found that the group with the top 64 features greatly improved the performance of our proposed model. Moreover, to thoroughly investigate the instant-based analysis of the extracted features, we performed LIME analysis on the randomly selected instance after SHAP analysis to predict the targeted classes, as shown in Fig. 3. Where class 1 represents the positive class and class 0 represents the negative class.

### Samples visualization via tSNE

**Fig. 4:**
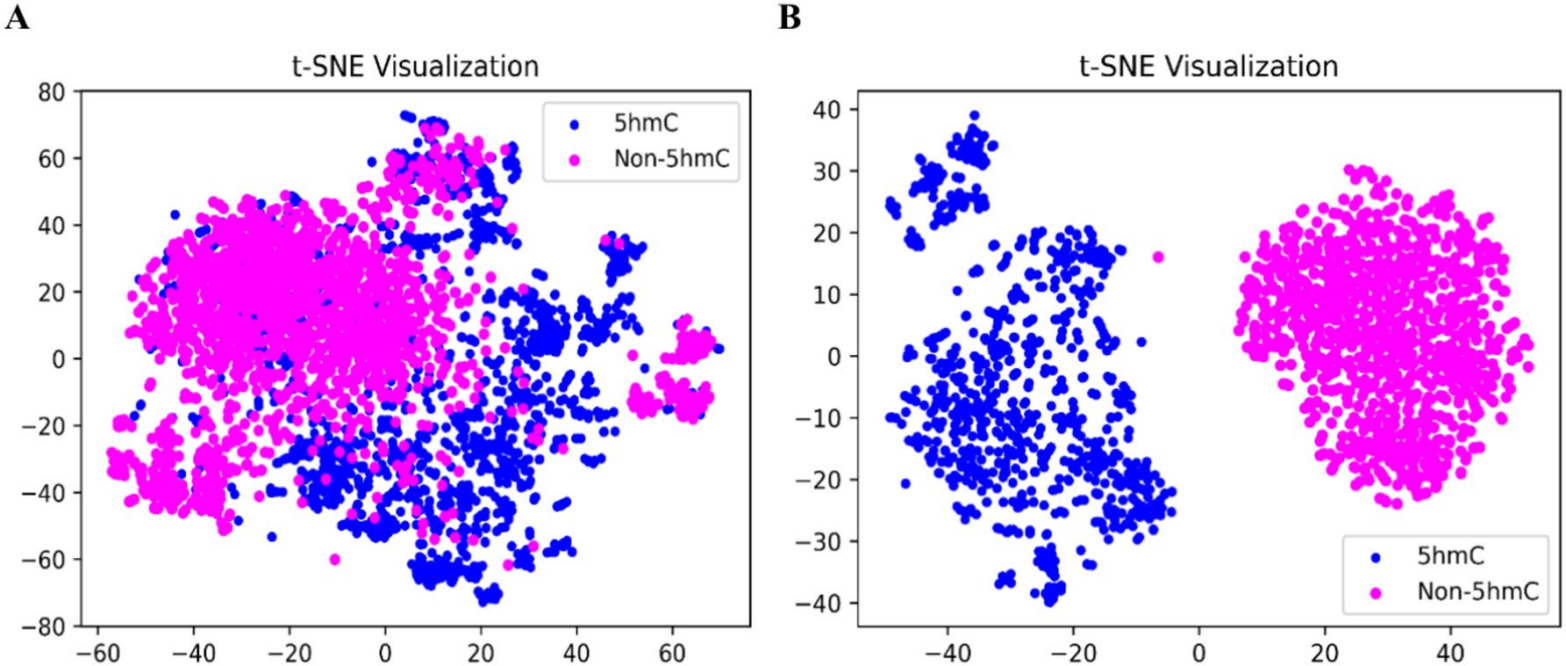
(**A**) t-SNE visualization of (**A**) Hybrid Training features (**B**) SHAP-based Selected features. This figure shows the t-SNE visualization for two feature sets: (**A**) hybrid training features and (**B**) SHAP-based selected features. The visualization helps illustrate the clustering and separation of data points in different feature spaces.

In order to investigate the effects of the extracted features, we used t-distributed Stochastic Neighbor Embedding (t-SNE) based feature visualization for the hybrid feature vector before and after applying feature selection as shown in Fig. 4. t-SNE maps revealed distinct clusters, facilitating the differentiation of positive and negative samples within them. False negative and false positive samples were notably situated between true negative and true positive samples, though their occurrences were infrequent. tSNE approach represents the local structural information as well as the global structural relationships^39^. The extracted features are further visualized using the t-SNE approach to convert the high-dimension vector into 2D space, as shown in Fig. 4. In Fig. 4A, the hybrid features show some degree of overlap between positive and negative samples, which is somewhat effective but does not accurately classify the targeted classes. However, in Fig. 4B, the data samples of both classes are clearly separable, demonstrating the effectiveness of SHAP-based optimal features in predicting between 5hmc and non-5hmc compared to the hybrid features in Fig. 4A.

### XGBoost

**Fig. 5:**
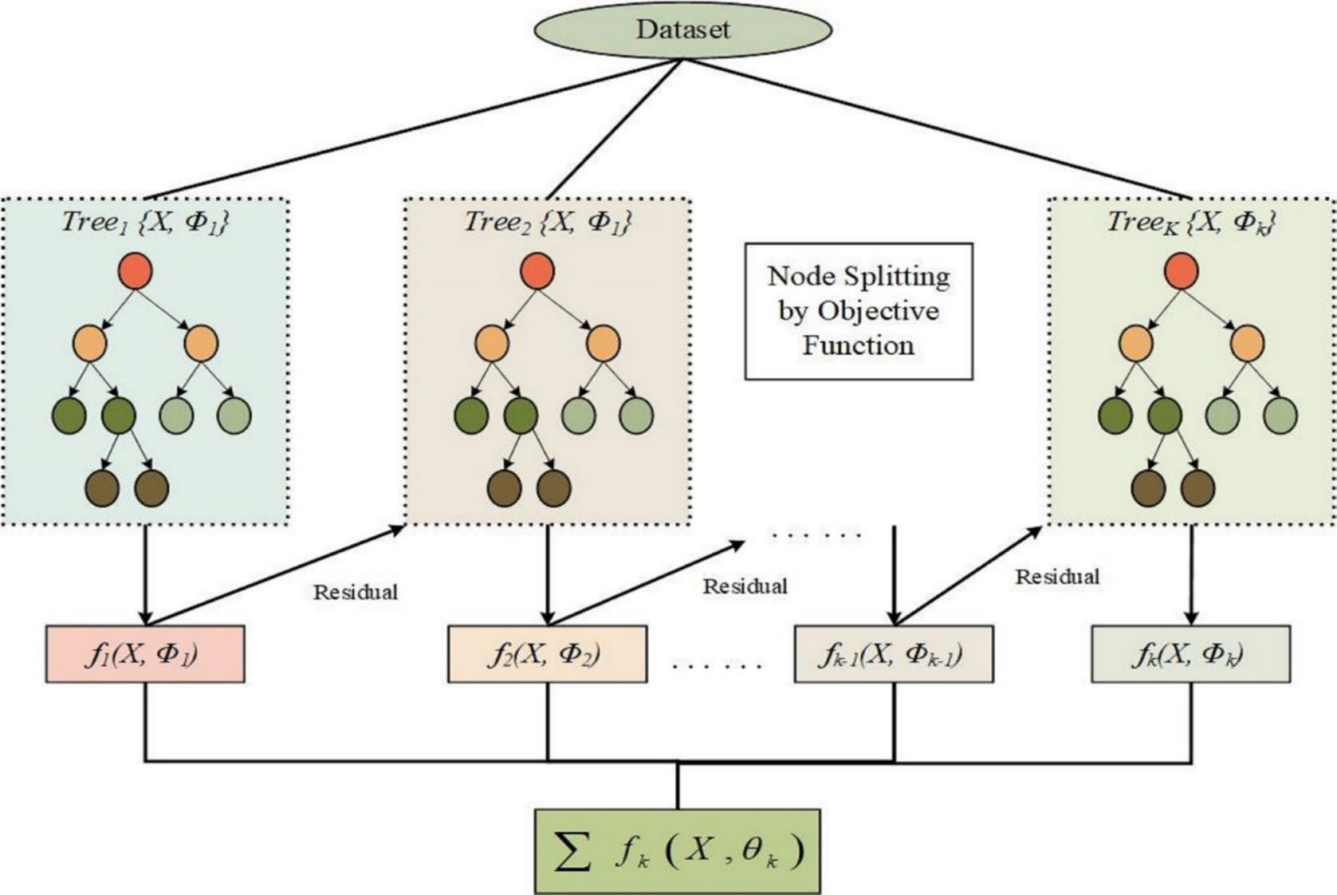
The Workflow of the XGBoost Algorithm. This figure depicts the step-by-step workflow of the XGBoost algorithm, including data input, boosting process, and final model output. It illustrates how the algorithm enhances prediction accuracy through iterative training and optimization.

XGBoost is defined as the extreme gradient boosting library. This optimized distributed gradient boosting library is meant for a faster, more flexible, and scalable machine learning process. At its core, the approach used by XGBoost is called gradient boosting, one of several ensemble learning algorithms that create a predictive model by weighing the results of several weak learners, usually decision trees^40,41^. XGBoost provides a class of matrix that is meant for the exclusively efficient performance of XGBoost functions in data storage and access during model training and evaluation. The goal of regression tasks in XGBoost is to minimize the mean squared error (MSE) between the then-observed and the forecast values. XGBoost loss functions consist of squared error, absolute error, and Huber loss. The objective and loss functions in the XGBoost tutorial describe the criteria for the training optimization process. We use the objective function to measure the model overall and the loss function to compare the predicted and actual values. There are two stages of XGBoost during which training and evaluation occur repeatedly by adding new trees into the sum of the gain function. Each tree is non-parallelly matched to the negative slope of the loss function that showed the incorrect answers of the previous trees. XGBoost validation through cross-validation is an approach for ascertaining model success. It implies dividing the dataset into several subsets and using varied combinations of those sets to train the model at each stage and estimate the quality level. When building an XGBoost classifier, obtaining some binary or multiclass classification objective function, such as logistic Regression or Softmax, is often necessary. Users can move from the native API of XGBoost to the one that is scikit-learn powered; this allows them to switch both ways without restriction, which means compatibility with other machine-learning libraries can be achieved. This lays out the path through which the users will enjoy the function of the two APIs with their specific choice, as shown in Fig. 5.

XGBoost works by constructing a set of decision trees that succeed one another and, in the end, make the predictions. It starts by assembling a dataset with the required input features (X) on one side and physical measurements (Φ) on the other. The structure is made up of decision trees called T1 to TK. The purpose of each tree is to rectify and correct the mistakes made by the previous tree. The algorithm computes the residuals of the previous model and subsequently fits a new tree in each iteration to minimize these residuals. This procedure continues until a certain amount of tree value (K) is reached. Then, the algorithm accumulates the separate models $\left( {f_{k} \left( {X, \, \Phi_{k} } \right)} \right)$ proposed by the individual trees to create a final prediction. Sequentially, it revises the model predictions addressing errors from the previous iteration, and finally, the approach delivers XGBoost highly accurate and robust forecasting for complex tasks.

XGBoost is an extreme gradient-boosting algorithm with various applications in machine learning, especially in regression and classification tasks. Know its efficiency, scalability, and high levels of predictive performance well. The logic is based on the concept of the tree ensemble, which represents an iterative process of building decision trees so that each tree rectifies the errors made by the previous ones. It optimizes a differentiable loss function with gradient descent, making it resilient and perform even in high-dimensional datasets. XGBoost main objective is to minimize the value of the loss function, which represents the divergence of the actual against the forecasted values. Here, several weak learners (decision trees), which are lightweight, are combined step-by-step into strong learners (ensemble model). The procedure sets the parameters of each tree as per the loss function gradient and gives them room to correct themselves over each iterative modification. The knowledge of the background and purposes of XGBoost constitutes a good base for studying the algorithmic details of the algorithm’s superior performance in the machine learning application.

**Table:** 

**XGBoost Algorithm: Start with a dataset containing input features** 1. **Initialize Model Parameters:** • Set hyperparameters: Learning rate: η • Maximum depth of trees: Number of trees: T 2. **Prepare Data:** • Load dataset: X (features) and y (labels). Split data into training and validation sets • Initialize ensemble: $\hat{f}^{0} \left( x \right) = mean\left( y \right)$ 3. **For each iteration (t):** • Compute negative gradient: $g_{i} = \frac{\partial }{{\partial \hat{f}^{t - 1} (x_{i} )}}L(y_{i} ,\hat{f}^{t - 1} (x_{i} ))$ • Compute second-order gradient (optional): $h_{i} = \frac{{\partial^{2} }}{{\partial \hat{f}^{t - 1} (x_{i} )^{2} }}L(y_{i} ,\hat{f}^{t - 1} (x_{i} ))$ • Train decision tree $h_{t}$​ to predict negative gradient:$h_{t} = \arg \min_{h} \sum\limits_{t = 1}^{N} {\left[ {g_{i} h(x_{i} ) + \frac{1}{2}h(x_{i} )^{2} + \Omega (h)} \right]}$ • Update model: $\hat{f}^{t} (x) = \hat{f}^{t - 1} (x) + \eta h_{t} (x)$ 4. **Regularization:** • Apply regularization techniques and tree pruning: $\Omega \left( h \right)$ • Feature subsampling: $\gamma$ 5. **Evaluation:** • Evaluate performance on validation set using evaluation metrics: $L(y,\hat{f}^{t} (x))$ 6. **Stopping Criteria:** • Check convergence or the predefined number of iterations: $t$ if converged or $t = T$ stop training 7. **Output Model:** • Return final ensemble of decision trees:$\hat{f}(x) = \sum\limits_{t = 1}^{T} {\eta h_{t} (x)}$	

## Performance evaluation matrix

The Performance Evaluation Matrix becomes the most precious tool for recognizing the working well or any flaws in the machine learning models^17,42^. It involves creating well-defined indicators to be used as model validation tools for assessing the performance in the different parameters (dimensions). Concision would be a plain yardstick of general correctness, while precision would weigh the model on false positives. The mind of the physician is called "Recall," which means the doctor’s ability to identify all relevant instances correctly. At the same time, "Specificity"(false positives) is his ability to recognize negative misdiagnoses accurately. F1 Score makes the presence felt equally considering both precision and recall, especially when dealing with imbalanced sample space. MCC (Matthews Correlation Coefficient) is relevant in imbalanced datasets with several actual positive, false-negative, true-negative, and false-positive values covering all the items in the confusion matrix^43–46^. Moreover, classifier discrimination and tau that use ROC Curve and AUC measure the model’s discriminatory power between classes. In contrast, the confusion matrix shows the number of correct and false predictions for each class. Alongside, these metrics offer a holistic evaluation tool that steers the way of the model to be more relevant to the current needs in machine learning technologies.

- *Accuracy:* Denotes the proportion of correctly classified objects regarding the number of total instances.
  14 $$Accuracy = \frac{TP + TN}{{TP + TN + FP + FN}}$$
- *Precision:* Shows the correct proportion of optimistic predictions made among all positive outcomes.
  15 $$Precision = \frac{TP}{{TP + FP}}$$
- *Sensitivity***: **On a ratio scale, how many correct predictions were made compared to the actual positive events that occurred.
  16 $$Recall = \frac{TP}{{TP + FN}}$$
- *Specificity***: **This indicator measures the portion of true negative classifications over all present negative cases.
  17 $$Specificity = \frac{TN}{{TN + FP}}$$
- *F1 Score:* Harmonic mean of precision and recall, its initial application on imbalanced datasets lays the groundwork for the emergence of more sophisticated methods.
  18 $$F1\,Score = 2 \times \frac{Precision \times Recall}{{Precision + Recall}}$$
- *Matthews Correlation Coefficient (MCC*): It was a perfect fit for balanced datasets with class imbalance as it touched all four confusion matrix metrics.
  19 $$MCC = \frac{TP \times TN - FP \times FN}{{\sqrt {(TP + FP)(TP + FN)(TN + FP)(TN + FN)} }}$$
  - *ROC Curve and AUC***: **The receiver operating characteristic (ROC) curve, based on true positive rate (Sensitivity) against false positive rate (1-Specificity), specifies the discriminant ability of the model; its area under the curve (AUC) is the quantification of this ability.
  - *Confusion Matrix***: **The Model is programmed and instructed to TP, TN, FP, and FN.

## Experimental results and analysis

In machine learning, to thoroughly evaluate the model the prediction outcomes of an intelligent model can be examined using several cross-validation tests. Among these tests, independent test, and k-fold subsampling test are commonly used to enhance the performance outcomes of a hypothesis learner. In this paper, we used rigorous evaluation techniques, including the k-fold (i.e. k = 10) cross-validation test and the independent dataset test, to thoroughly examine the DNN model’s performance. These methods were particularly effective in demonstrating the model’s effectiveness and generalization ability.

### Performance evaluation

In this section, we evaluate the prediction ability of the proposed model by applying various individual feature extraction techniques, including Mismatch, ANF, PSTNPss, ASDC, DAC, and composite features using different cross-validation tests. Table 1 presents the performance of the XGBoost model with various sequence formulation techniques, evaluated using fivefold cross-validation. We can observe from Table 1 that the XGBoost model yielded the best performance using hybrid features compared with other feature formulation methods. For instance, the XGBoost model achieved an average success rate of 85.61% before applying feature selection. In order to further improve the performance of the XGBoost model, the dimensionality of the hybrid features space was reduced using the feature selection method. As a result, the success rate of the XGBoost model was significantly improved i.e. an average accuracy improved to 86.43% as shown in Table 1.

**Table 1:** Performance evaluation of XGBoost model using fivefold cross-validation.

Method	ACC (%)	SN (%)	Precision (%)	SPE (%)	F1 Score (%)	MCC	AUC
Mismatch	82.35	75.21	78.93	82.46	71.34	0.573	0.813
ANF	83.14	68.34	76.83	84.75	73.05	0.612	0.837
PSTNPss	83.57	70.65	79.28	86.08	75.75	0.652	0.855
ASDC	84.26	75.03	82.48	88.85	80.92	0.724	0.884
DAC	85.18	80.72	85.58	90.57	83.32	0.785	0.912
Hybrid feature (before feature selection)	85.61	82.12	87.52	92.75	85.41	0.814	0.932
Hybrid feature (after feature selection)	86.43	82.35	84.31	90.12	86.71	0.821	0.938

Table 2 compares the accuracy gains of individual and hybrid features on the XGBoost classifier using a tenfold cross-validation test. We can see from Table 2, that the XGBoost model also obtained the highest performance using hybrid features compared with other sequence formulation methods. For example, the XGBoost model achieved average accuracy (i.e. 86.87%) before applying the feature selection method. Similarly, the success rate of the XGBoost model further improved (i.e. 89.97%) using the feature selection method as shown in Table 2. These results confirmed that the XGBoost model generates the best prediction performance on hybrid features using tenfold cross-validation.

**Table 2:** Performance evaluation of XGBoost model using tenfold cross-validation.

Method	ACC (%)	SN (%)	Precision (%)	SPE (%)	F1 Score (%)	MCC	AUC
Mismatch	75.13	65.21	78.93	82.46	71.34	0.573	0.813
ANF	77.34	68.34	76.83	84.75	73.05	0.612	0.837
PSTNPss	78.09	70.65	79.28	86.85	75.75	0.652	0.855
ASDC	82.94	75.03	82.48	88.85	80.92	0.724	0.884
DAC	85.95	80.72	85.58	90.57	83.32	0.785	0.912
Hybrid feature (before feature selection)	86.87	82.12	87.52	92.75	85.41	0.814	0.932
Hybrid feature (after feature selection)	89.97	87.78	90.94	94.45	89.34	0.876	0.953

**Fig. 6:**
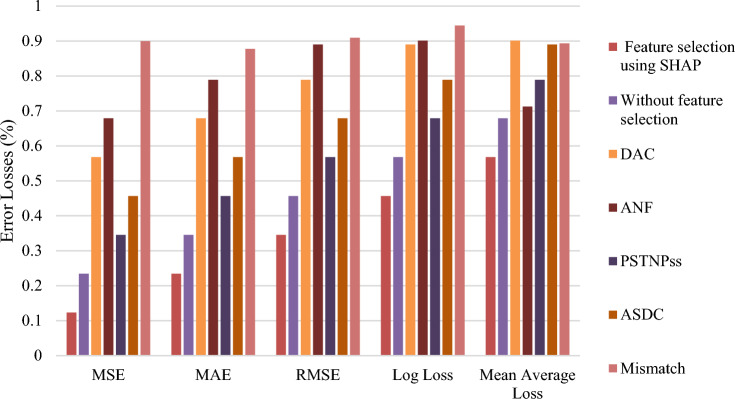
Performance Evaluation of XGBoost Model with Different Sequence Formulation Techniques and Error Metrics. This figure compares the performance of the XGBoost model using various sequence formulation techniques, highlighting key error metrics. It visualizes how different techniques affect the model’s accuracy, precision, and other performance measures.

In addition to the performance metrics, Fig. 6 presents the performance of the XGBoost model using various sequence formulation techniques, measured by different error metrics. Each technique represents different error measures, including Mean Squared Error (MSE), Mean Absolute Error (MAE), Root Mean Squared Error (RMSE), Log Loss, and Mean Average Loss. Using these metrics together ensures a comprehensive evaluation of the model, capturing both the average performance and the impact of larger errors or misclassifications. For all these metrics, lower values indicate better model performance, as they signify smaller errors between the predicted and actual values. For instance, the feature selection using the SHAP technique achieves the best results with the lowest values in all metrics: MSE of 0.1234, MAE of 0.2345, RMSE of 0.3456, log loss of 0.4567, and mean average loss of 0.5678. In comparison, the without-feature selection approach shows increased values, reflecting a decline in performance, with MSE of 0.2345, MAE of 0.3456, RMSE of 0.4567, log loss of 0.5678, and mean an average loss of 0.6789.

### Analysis of different learning classifiers

In this section, we compare the performance of the proposed model with other widely used machine learning algorithms using hybrid features. For the performance comparison, we considered the classifiers including Random Forest (RF), Support-Vector-Machine (SVM), K-Nearest Neighbor (KNN), Naive Bayes (NB), and Logistic Regression (LR). RF is an ensemble learning algorithm commonly used for different classification and regression problems^47^. It applies a bootstrap algorithm to construct multiple decision trees based on random selection using training samples^20,35^. KNN is an instance-based and non-parametric learning algorithm commonly used in the area of image processing. It measures Euclidian distance amongst the instances for classification purposes^19^. SVM is a powerful classification algorithm and is widely used in the area of bioinformatics^48^. It is used for both linear and non-linear classification problems. It computes optimal hyper-plane to differentiate between the classes^17^. NB is a simple yet powerful probabilistic classifier based on Bayes’ theorem, assuming independence between features. It is highly efficient for large datasets and performs well with text classification tasks like spam detection. Despite its simplicity, Naive Bayes can achieve surprisingly high accuracy in various applications^37,49–53^. LR is a widely used statistical method for binary classification, which models the probability of a binary outcome using a logistic function. It estimates the relationship between the dependent binary variable and one or more independent variables by using maximum likelihood estimation. Logistic Regression is popular due to its simplicity, interpretability, and effectiveness in many practical applications, including medical diagnosis and credit scoring^54^. The optimal parameters used for the training these models are provided in Table 3. The performance comparison of various algorithms is provided in Table 4. According to Table 4, XGBoost demonstrated the highest accuracy at 89.97%, outperforming all other classifiers across all measures. The Random Forest (RF) algorithm achieved the next highest accuracy at 82.56%. In terms of the Matthews Correlation Coefficient (MCC), which represents the stability of a model, the XGBoost model achieved the highest value of 0.876, compared to the next highest value of 0.698 achieved by the RF algorithm.

**Table 3:** Optimal parameters used for the machine learning models.

Classifier	Parameter	Value
XGBoost	n_estimators	200
	Learning rate	0.01
	Max depth	20
	Min child weight	10
	gamma	0.5
	Booster	Gbtree
	Objective Function	Binary logistics
	Col sample by level	0.5, 0.8, 1.0
	lambda (reg_alpha)	0.1, 1
	alpha (reg_lambda)	0.1, 1
	Random state	42
	n_estimators	200
RF	n_estimators	200
	Bootstrap	True
	Random state	42
	Criterion	Entropy
	Max_features	Auto
	Max_depth	20
	min_samples_split	9
	min_samples_leaf	5
SVM	Gamma	0.001
	Kernal	RBF
	C	15
	Random state	42
KNN	Nearest neighbors	11
	Random state	42

**Table 4:** Comparative analysis of learning classifiers.

Classifier	ACC (%)	SN (%)	Precision (%)	SPE (%)	F1 score (%)	MCC	AUC
XGBoost	89.97	87.78	90.94	94.45	89.34	0.876	0.953
RF	82.56	74.23	81.56	87.94	78.23	0.698	0.865
KNN	81.23	72.96	80.52	86.93	77.34	0.689	0.855
SVM	80.12	71.45	79.85	85.79	76.12	0.673	0.845
NB	79.54	71.12	79.12	85.47	74.56	0.667	0.838
LR	78.89	69.87	78.54	84.56	73.21	0.651	0.832

### Analysis of learning hypotheses using independent dataset

We extensively evaluated using an independent dataset to ensure our groundbreaking model’s highest stability and unwavering reliability. The highly detailed and meticulously organized results in Table 5 provide a profound understanding of the hybrid feature set’s remarkable performance across diverse classifiers. It is especially noteworthy that our pioneering proposed classifier demonstrated unparalleled accuracy, achieving an astounding 84.87%. Furthermore, it outperformed all other classifiers by performing an amazing 77.76% sensitivity and an exceptional 90.34% specificity. In addition, on the composite feature set, the Random forest classification served well, attaining the second-highest accuracy and special MCC values of 82.53 and 0.698 respectively.

**Table 5:** Performance evaluation of learning hypotheses using independent dataset.

Classifier	ACC (%)	SN (%)	Precision (%)	SPE (%)	F1 Score (%)	MCC	AUC
XGBoost	84.87	77.76	84.32	90.34	81.56	0.741	0.888
RF	82.53	74.24	81.67	87.45	78.32	0.698	0.862
SVM	83.45	75.43	82.74	88.86	79.12	0.716	0.875
NB	82.32	78.20	80.54	84.41	81.32	0.708	0.864
LR	81.23	73.32	80.32	86.94	77.23	0.688	0.853
KNN	80.56	72.21	79.83	85.95	75.56	0.672	0.845

### The XGBoost model of the performance visualization

**Fig. 7:**
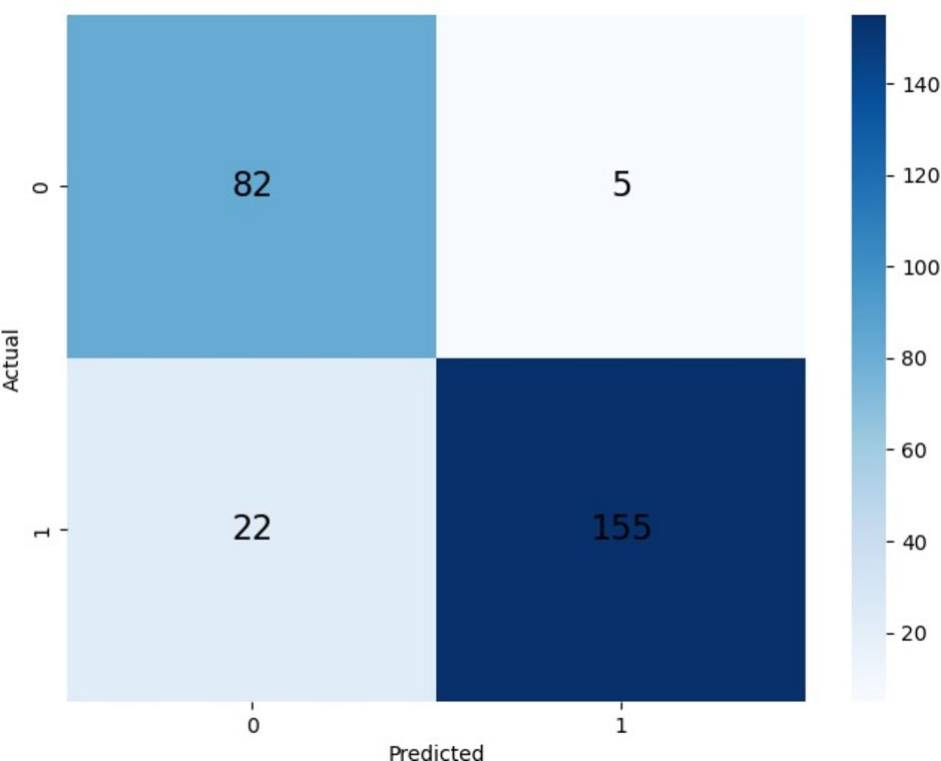
Confusion Matrix of the XGBoost Model using Training subset. This figure displays the confusion matrix for the XGBoost model evaluated on the training subset, showing the number of true positives, true negatives, false positives, and false negatives to assess the model’s classification performance.

The predicted performance of the XGBoost model is also visually interpreted via a confusion matrix as shown in Fig. 7. We employed a train/test split of the benchmark dataset. The benchmark dataset is divided into two parts: 80% of the samples are allocated for training, while 20% are reserved for testing (validation data). The validation data of 20% were randomly selected to investigate the training model. To thoroughly assess the model generalization and overfitting, we evaluate the validation data by providing the predicted outcomes in the form of a confusion matrix.

**Fig. 8:**
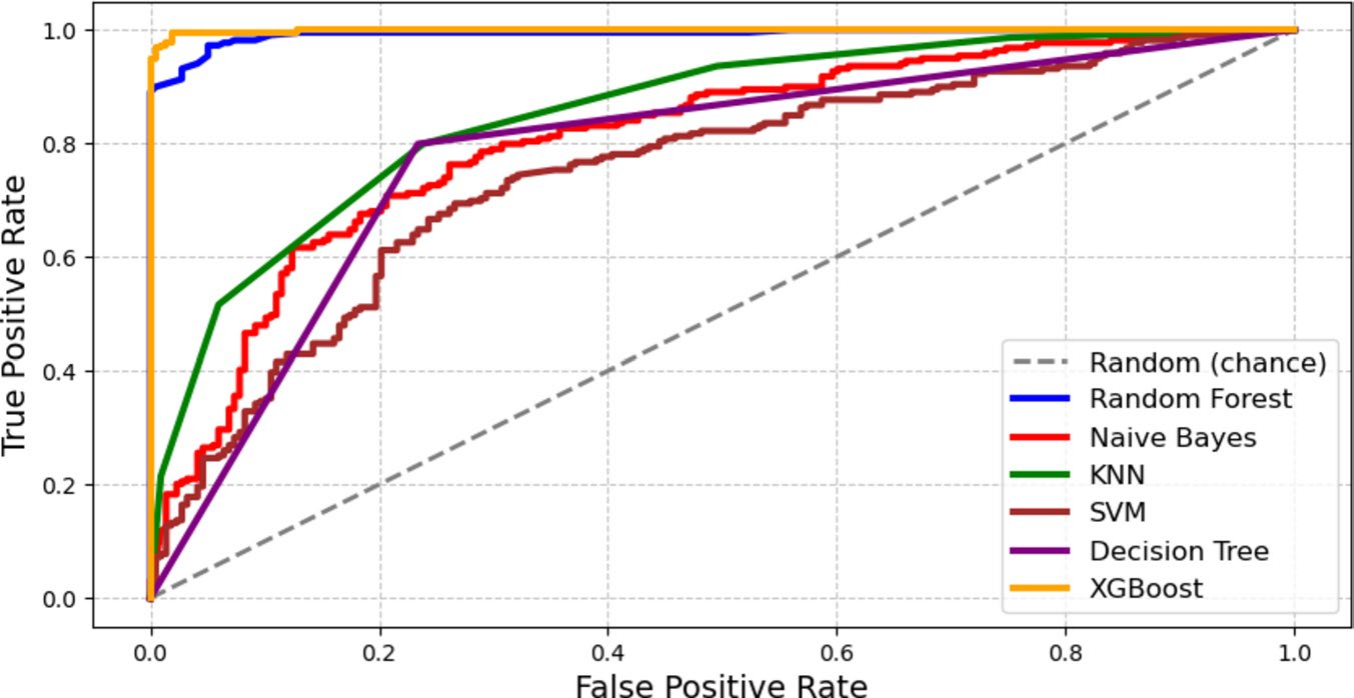
Comparative Performance Analysis of Machine Learning Models. This figure presents a comparative analysis of different machine learning models, highlighting their performance metrics such as accuracy, precision, and recall. It illustrates how each model, including XGBoost, fares in predicting outcomes and helps in evaluating their relative effectiveness.

When comparing the performance of different machine learning models such as XGBoost, Random Forest, Support Vector Machine, Logistic Regression, and K-Nearest Neighbors. XGBoost consistently outperforms the rest in visualizations. In particular, when comparing the ROC curves, XGBoost performs better using various evaluation metrics. Its curve consistently shows a greater area under the curve (AUC) values, suggesting an improved ability to discriminate and predict outcomes. Figure 8 demonstrates the efficiency of XGBoost in managing intricate data sets and its ability to surpass other standard machine learning algorithms.

### Comparison of performance of previous methods with the current models

To determine the efficacy of the current study, we compared the novel predictor with existing models. Table 6 presents the results for identifying the 5hmC sites, comparing the novel predictor with existing methods, including iRNA5hmC employing SVM, iRNA5hmC-PS utilizing LR, and iRhm5CNN with CNN. Evaluation metrics such as Accuracy (ACC), Sensitivity (SN), Specificity (SP), F1 Score, and Matthews Correlation Coefficient (MCC) were utilized. From Table 5, the iRNA5hmC model achieved a 65.48% accuracy, 67.67% sensitivity, 63.29% specificity, and a 65.01% F1 Score, but had a relatively low MCC of 0.31. The iRNA5hmC-PS model improved performance with a 78.3% accuracy, increased sensitivity, specificity, and F1 Score, and a moderate MCC of 0.56. The iRhm5CNN model showed even better results, achieving 81% accuracy, balanced sensitivity and specificity, and a higher MCC of 0.62. The XGBoost5hmC model excelled with an accuracy of 89.97%, 87.78% sensitivity, 94.45% specificity, 89.34% F1 Score, and a robust MCC of 0.876.

**Table 6:** Performance comparison with existing models.

Methods	ACC (%)	SN (%)	SP (%)	F1 Score (%)	MCC
iRNA5hmC^14^	65.48	67.67	63.29	0.6501	0.310
iRNA5hmC-PS^15^	78.30	80.00	79.50	76.40	0.560
iRhm5CNN^16^	81.00	82.00	80.00	78.00	0.620
XGBoost5hmC	89.97	87.78	94.45	89.34	0.876

The overall experimental results demonstrated that the proposed XGBoost model can effectively detect the presence of 5hmC sites. Throughout the evaluation and comparison with other machine learning methods, XGBoost consistently outperformed the others in terms of accuracy, sensitivity, precision, specificity, F1 score, Matthew’s correlation coefficient, and area under the curve. The theoretical approach of constructing syntactic models using various sequential creation methods enhanced the model’s performance by incorporating a mix of hybrid features and feature selection, thereby improving prediction accuracy. A comparison of different methods identifies the XGBoost5hmC model as the most significant enhancer, surpassing all others in accuracy, sensitivity, specificity, F1 score, and MCC. The model’s effectiveness and reliability were confirmed using a separate fold with tenfold cross-validation. The results indicate that the XGBoost model is feasible and valuable for accurately detecting 5hmC sites, providing crucial insights for future research and practical applications in bioinformatics and epigenetics.

## Conclusion

This study design for XGB5hmC indicates the achievement of a critical goal in improved detection of the RNA modification species, and the particular species being logic here is the 5-hydroxymethylcytosine (5hmC). XGBoost, a known strong gradient boosting method favored by discriminative feature extraction, performs better in identifying 5hmC sites than the current models. The proposed XGB5hmC model using k-fold test achieved the predicted accuracy of 89.97%, demonstrating its effectiveness and reliability. Furthermore, the model highlights RNA modification maps’ complexities by revealing gene expression regulatory ways and epigenetic control systems. The role of RNA modification understanding would undergo profound changes with the ability of RNAseq to predict the 5hmC alteration pattern precisely. The discovery of novel biological processes and their impacts on human health can be possible. The diversity of the XGB5hmc model is evident in its arsenal of hybrid features and advanced machine-learning approaches. The model is promising for the early diagnosis of the disease, and it has a very high impact on many areas, mainly cancer, diabetes, and the cardiovascular system. The XGB5hmC method contributes to the scientific community by providing a systematic approach for RNA modifications that promotes biomedicine through advanced knowledge and enhances the chances of designing customized therapies. The XGB5hmC model provides a breakthrough in the investigation of RNA modifications. This study can potentially disclose how genes develop regulatory roles and contribute to various diseases.

## Data Availability

The datasets used and/or analyzed during the current study are available on Github link https://github.com/salman-khan-mrd/5HMC-2.

## References

[1] Brosius, J. & Raabe, C. A. What is an RNA? A top layer for RNA classification. *RNA Biol.* **13**, 140–144 (2016). 26818079 10.1080/15476286.2015.1128064 PMC4829331

[2] Igamberdiev, A. U. & Kleczkowski, L. A. Toward understanding the emergence of life: A dual function of the system of nucleotides in the metabolically closed autopoietic organization. *Biosystems* **224**, 104837 (2023). 36649884 10.1016/j.biosystems.2023.104837

[3] Chen, Y. *et al.* The functions and mechanisms of post-translational modification in protein regulators of RNA methylation: Current status and future perspectives. *Int. J. Biol. Macromol.* **253**, 126773 (2023). 37690652 10.1016/j.ijbiomac.2023.126773

[4] Van Deuren, V., Plessers, S. & Robben, J. Structural determinants of nucleobase modification recognition in the AlkB family of dioxygenases. *DNA Repair* **96**, 102995 (2020). 33069898 10.1016/j.dnarep.2020.102995

[5] Fitzsimmons, C. M. *et al.* Rewiring of RNA methylation by the oncometabolite fumarate in renal cell carcinoma. *NAR Cancer* **6**, zcae004 (2024). 38328795 10.1093/narcan/zcae004 PMC10849186

[6] Lio, C.-W.J. *et al.* TET methylcytosine oxidases: New insights from a decade of research. *J. Biosci.* **45**, 1–14 (2020). 31965999 10.1007/s12038-019-9973-4 PMC7216820

[7] Bi, D., Almpanis, A., Noel, A., Deng, Y. & Schober, R. A survey of molecular communication in cell biology: Establishing a new hierarchy for interdisciplinary applications. *IEEE Commun. Surv. Tutor.* **23**, 1494–1545 (2021). 10.1109/COMST.2021.3066117

[8] Fu, L. *et al.* Tet-mediated formation of 5-hydroxymethylcytosine in RNA. *J. Am. Chem. Soc.* **136**, 11582–11585 (2014). 25073028 10.1021/ja505305z PMC4140497

[9] Huber, S. M. *et al.* Formation and abundance of 5-hydroxymethylcytosine in RNA. *Chembiochem* **16**, 752–755 (2015). 25676849 10.1002/cbic.201500013 PMC4471624

[10] Everroad, R. C. *et al.* Space Biology Beyond LEO Instrumentation & Science Series-Science Working Group 2021 Annual Report (2021).

[11] Roundtree, I. A., Evans, M. E., Pan, T. & He, C. Dynamic RNA modifications in gene expression regulation. *Cell* **169**, 1187–1200 (2017). 28622506 10.1016/j.cell.2017.05.045 PMC5657247

[12] Uribe-Lewis, S. *et al.* 5-hydroxymethylcytosine and gene activity in mouse intestinal differentiation. *Sci. Rep.* **10**, 546 (2020). 31953501 10.1038/s41598-019-57214-z PMC6969059

[13] Dong, Z.-W. *et al.* RTL-P: A sensitive approach for detecting sites of 2′-*O*-methylation in RNA molecules. *Nucleic Acids Res.* **40**, e157–e157 (2012). 22833606 10.1093/nar/gks698 PMC3488209

[14] Liu, Y., Chen, D., Su, R., Chen, W. & Wei, L. iRNA5hmC: The first predictor to identify RNA 5-hydroxymethylcytosine modifications using machine learning. *Front. Bioeng. Biotechnol.* **8**, 227 (2020). 32296686 10.3389/fbioe.2020.00227 PMC7137033

[15] Ahmed, S. *et al.* Accurate prediction of RNA 5-hydroxymethylcytosine modification by utilizing novel position-specific gapped k-mer descriptors. *Comput. Struct. Biotechnol. J.* **18**, 3528–3538 (2020). 33304452 10.1016/j.csbj.2020.10.032 PMC7701324

[16] Ali, S. D., Kim, J. H., Tayara, H. & to Chong, K. Prediction of rna 5-hydroxymethylcytosine modifications using deep learning. *IEEE Access* **9**, 8491–8496 (2021). 10.1109/ACCESS.2021.3049146

[17] Khan, S. *et al.* Sequence based model using deep neural network and hybrid features for identification of 5-hydroxymethylcytosine modification. *Sci. Rep.* **14**, 9116 (2024). 38643305 10.1038/s41598-024-59777-y PMC11551160

[18] Khan, S., Khan, M., Iqbal, N., Khan, S. A. & Chou, K.-C. Prediction of piRNAs and their function based on discriminative intelligent model using hybrid features into Chou’s PseKNC. *Chemom. Intell. Lab. Syst.* **203**, 104056 (2020). 10.1016/j.chemolab.2020.104056

[19] Khan, S., Khan, M., Iqbal, N., Rahman, M. A. A. & Karim, M. K. A. Deep-PiRNA: Bi-layered prediction model for PIWI-interacting RNA using discriminative features. *Comput. Mater. Contin.* **72**, 2243–2258 (2022).

[20] Naeem, M. & Qiyas, M. Deep intelligent predictive model for the identification of diabetes. *AIMS Math.* **8**, 16446–16462 (2023). 10.3934/math.2023840

[21] Luo, L. *et al.* Accurate prediction of transposon-derived piRNAs by integrating various sequential and physicochemical features. *PloS One* **11**, e0153268 (2016). 27074043 10.1371/journal.pone.0153268 PMC4830532

[22] Carlile, T. M., Rojas-Duran, M. F. & Gilbert, W. V. *Methods in Enzymology* Vol. 560, 219–245 (Elsevier, 2015). 10.1016/bs.mie.2015.03.011 PMC7945874 26253973

[23] Chen, W., Tran, H., Liang, Z., Lin, H. & Zhang, L. Identification and analysis of the N6-methyladenosine in the Saccharomyces cerevisiae transcriptome. *Sci. Rep.* **5**, 13859 (2015). 26343792 10.1038/srep13859 PMC4561376

[24] Chen, Z. *et al.* Comprehensive review and assessment of computational methods for predicting RNA post-transcriptional modification sites from RNA sequences. *Brief. Bioinf.* **21**, 1676–1696 (2020). 10.1093/bib/bbz112 31714956

[25] Cursons, J. *et al.* Combinatorial targeting by microRNAs co-ordinates post-transcriptional control of EMT. *Cell Syst.* **7**, 77–91 (2018). 30007539 10.1016/j.cels.2018.05.019

[26] Xuan, J.-J. *et al.* RMBase v2.0: Deciphering the map of RNA modifications from epitranscriptome sequencing data. *Nucleic Acids Res.* **46**, D327–D334 (2018). 29040692 10.1093/nar/gkx934 PMC5753293

[27] Khan, S., Khan, M., Iqbal, N., Li, M. & Khan, D. M. Spark-based parallel deep neural network model for classification of large scale RNAs into piRNAs and non-piRNAs. *IEEE Access* **8**, 136978–136991 (2020). 10.1109/ACCESS.2020.3011508

[28] Khan, S. *et al.* Optimized feature learning for anti-inflammatory peptide prediction using parallel distributed computing. *Appl. Sci.* **13**, 7059 (2023). 10.3390/app13127059

[29] Liu, Q. *et al.* DeepTorrent: a deep learning-based approach for predicting DNA N4-methylcytosine sites. *Brief. Bioinf.* **22**, bbaa124 (2021). 10.1093/bib/bbaa124 PMC8599298 32608476

[30] Wei, L., Zhou, C., Chen, H., Song, J. & Su, R. ACPred-FL: a sequence-based predictor using effective feature representation to improve the prediction of anti-cancer peptides. *Bioinformatics* **34**, 4007–4016 (2018). 29868903 10.1093/bioinformatics/bty451 PMC6247924

[31] Wei, L., Tang, J. & Zou, Q. SkipCPP-Pred: an improved and promising sequence-based predictor for predicting cell-penetrating peptides. *BMC Genom.* **18**, 1–11 (2017). 10.1186/s12864-017-4128-1 PMC5657092 29513192

[32] Liu, B., Liu, F., Fang, L., Wang, X. & Chou, K.-C. repDNA: A Python package to generate various modes of feature vectors for DNA sequences by incorporating user-defined physicochemical properties and sequence-order effects. *Bioinformatics* **31**, 1307–1309 (2015). 25504848 10.1093/bioinformatics/btu820

[33] Lin, H., Deng, E.-Z., Ding, H., Chen, W. & Chou, K.-C. iPro54-PseKNC: A sequence-based predictor for identifying sigma-54 promoters in prokaryote with pseudo k-tuple nucleotide composition. *Nucleic Acids Res.* **42**, 12961–12972 (2014). 25361964 10.1093/nar/gku1019 PMC4245931

[34] Khan, F. *et al.* Prediction of recombination spots using novel hybrid feature extraction method via deep learning approach. *Front. Genet.* **11**, 539227 (2020). 33093842 10.3389/fgene.2020.539227 PMC7527634

[35] Khan, S. *et al.* Enhancing sumoylation site prediction: A deep neural network with discriminative features. *Life* **13**, 2153 (2023). 38004293 10.3390/life13112153 PMC10672286

[36] Heuillet, A., Couthouis, F. & Díaz-Rodríguez, N. Collective explainable AI: Explaining cooperative strategies and agent contribution in multiagent reinforcement learning with shapley values. *IEEE Comput. Intell. Mag.* **17**, 59–71 (2022). 10.1109/MCI.2021.3129959

[37] Akbar, S. *et al.* Prediction of antiviral peptides using transform evolutionary & SHAP analysis based descriptors by incorporation with ensemble learning strategy. *Chemom. Intel. Lab. Syst.* **230**, 104682 (2022). 10.1016/j.chemolab.2022.104682

[38] Raza, A. *et al.* AIPs-SnTCN: Predicting anti-inflammatory peptides using fastText and transformer encoder-based hybrid word embedding with self-normalized temporal convolutional networks. *J. Chem. Inf. Model.* **63**, 6537–6554 (2023). 37905969 10.1021/acs.jcim.3c01563

[39] Arif, R., Kanwal, S., Ahmed, S. & Kabir, M. Computational predictor for accurate identification of tumor homing peptides by integrating sequential and deep BiLSTM features. *Interdiscip. Sci. Comput. Life Sci.* **16**, 503–518. 10.1007/s12539-024-00628-9 (2024). 10.1007/s12539-024-00628-9 38733473

[40] Kavzoglu, T. & Teke, A. Predictive performances of ensemble machine learning algorithms in landslide susceptibility mapping using random forest, extreme gradient boosting (XGBoost) and natural gradient boosting (NGBoost). *Arabian J. Sci. Eng.* **47**, 7367–7385 (2022). 10.1007/s13369-022-06560-8

[41] Arshad, F., Ahmed, S., Amjad, A. & Kabir, M. An explainable stacking-based approach for accelerating the prediction of antidiabetic peptides. *Anal. Biochem.* **691**, 115546. 10.1016/j.ab.2024.115546 (2024). 38670418 10.1016/j.ab.2024.115546

[42] Ullah, M., Akbar, S., Raza, A. & Zou, Q. DeepAVP-TPPred: Identification of antiviral peptides using transformed image-based localized descriptors and binary tree growth algorithm. *Bioinformatics* **40**, btae305 (2024). 38710482 10.1093/bioinformatics/btae305 PMC11256913

[43] Akbar, S. *et al.* pAtbP-EnC: Identifying anti-tubercular peptides using multi-feature representation and genetic algorithm based deep ensemble model. *IEEE Access* (2023).

[44] Akbar, S., Zou, Q., Raza, A. & Alarfaj, F. K. iAFPs-Mv-BiTCN: Predicting antifungal peptides using self-attention transformer embedding and transform evolutionary based multi-view features with bidirectional temporal convolutional networks. *Artif. Intell. Med.* **151**, 102860 (2024). 38552379 10.1016/j.artmed.2024.102860

[45] Akbar, S., Raza, A. & Zou, Q. Deepstacked-AVPs: Predicting antiviral peptides using tri-segment evolutionary profile and word embedding based multi-perspective features with deep stacking model. *BMC Bioinf.* **25**, 102 (2024). 10.1186/s12859-024-05726-5 PMC10921744 38454333

[46] Raza, A. *et al.* Comprehensive analysis of computational methods for predicting anti-inflammatory peptides. *Arch. Comput. Methods Eng.* 1–19 (2024).

[47] Gill, M., Ahmed, S., Kabir, M. & Hayat, M. A novel predictor for the analysis and prediction of enhancers and their strength via multi-view features and deep forest. *Information*10.3390/info14120636 (2023). 10.3390/info14120636

[48] Ahmed, S., Kabir, M., Arif, M., Ali, Z. & Swati, Z. N. Prediction of human phosphorylated proteins by extracting multi-perspective discriminative features from the evolutionary profile and physicochemical properties through LFDA. *Chemom. Intell. Lab. Syst.* **203**, 104066. 10.1016/j.chemolab.2020.104066 (2020). 10.1016/j.chemolab.2020.104066

[49] Akbar, S. *et al.* iHBP-DeepPSSM: Identifying hormone binding proteins using PsePSSM based evolutionary features and deep learning approach. *Chemom. Intell. Lab. Syst.* **204**, 104103 (2020). 10.1016/j.chemolab.2020.104103

[50] Akbar, S. *et al.* iAtbP-Hyb-EnC: Prediction of antitubercular peptides via heterogeneous feature representation and genetic algorithm based ensemble learning model. *Comput. Biol. Med.* **137**, 104778 (2021). 34481183 10.1016/j.compbiomed.2021.104778

[51] Ahmad, A. *et al.* Identification of antioxidant proteins using a discriminative intelligent model of k-space amino acid pairs based descriptors incorporating with ensemble feature selection. *Biocybern. Biomed. Eng.* **42**, 727–735 (2022). 10.1016/j.bbe.2020.10.003

[52] Ahmad, A. *et al.* Deep-AntiFP: Prediction of antifungal peptides using distanct multi-informative features incorporating with deep neural networks. *Chemom. Intell. Lab. Syst.* **208**, 104214 (2021). 10.1016/j.chemolab.2020.104214

[53] Akbar, S., Hayat, M., Tahir, M., Khan, S. & Alarfaj, F. K. cACP-DeepGram: Classification of anticancer peptides via deep neural network and skip-gram-based word embedding model. *Artif. Intell. Med.* **131**, 102349 (2022). 36100346 10.1016/j.artmed.2022.102349

[54] Chiwanga, F. S. *et al.* Urban and rural prevalence of diabetes and pre-diabetes and risk factors associated with diabetes in Tanzania and Uganda. *Global health action* **9**, 31440 (2016). 27221531 10.3402/gha.v9.31440 PMC4879179

